# Bioactive glass putty with and without platelet-rich fibrin for periodontal intrabony defects: A prospective comparative study

**DOI:** 10.6026/973206300212669

**Published:** 2025-08-31

**Authors:** Lakshmi Narayanan S., Kennedy Babu S.P.K., Gandhimadhi D., Soorya K.V., Manoj M.

**Affiliations:** 1 Department of Periodontology, Mahatma Gandhi Postgraduate Institute of Dental Sciences, Gorimedu, Puducherry, India

**Keywords:** Periodontitis, intrabony defects, platelet rich fibrin, bioactive glass putty, periodontal regeneration

## Abstract

Periodontal treatment aims to halt the advancement of tissue destruction on one hand and promote the regeneration of lost periodontal structures on the other hand. Bioactive glass, an alloplastic bone graft material, supports bone regeneration by enhancing the formation of mineralized extracellular matrix through its adsorptive properties and by providing proteins that aid osteoblast function. Platelet-rich fibrin (PRF), a self - derived concentrate having growth factors and leukocytes, can significantly influence the cellular processes involved in periodontal regeneration. The fusion of bioactive glass and PRF may offer an effective approach for treating intrabony defects. In a split-mouth study design, 32 intraosseous defects were treated with either Novabone putty (Control site) or a combination of Novabone putty and PRF (Test site). Documentation of clinical variables was done at baseline, 3, 6, and 9 months after surgery and radiographic bone levels were also evaluated at the postsurgical visits. A significant decrease in PPD and an increase in CAL, relative to baseline measurements were observed in both groups. Attachment level showed modest enhancements in test groups. After 9 months, the test group exhibited a greater amount of bone fill compared to the control group. Thus, autologous PRF combined with bioactive glass bone graft (Novabone putty) has proven to be effective in enhancing both clinical and radiographic results for periodontal intrabony defects and offers additional advantages when compared to bone grafts by them.

## Background:

Periodontitis is a long-standing immunoinflammatory disease that ends with the destruction of the structural components anchoring the tooth to the jawbone that is periodontium. The primary consequence of this tissue breakdown, especially involving the alveolar bone, is tooth loss. Bone tissue homeostasis relies on a delicate balance between resorption and formation, which is disrupted during periodontal disease when resorptive activity surpasses bone formation. This excessive bone loss alters the typical structural morphology of bone. Pritchard (1965) identified several types of bone defects associated with periodontal disease, including interdental craters, uneven bone margins, intrabony defects, furcation involvements, or a mix of these patterns [1]. Ultimately, all periodontal treatments focus on restoring the lost attachment structures of teeth. As per Melcher, the regeneration of the periodontal ligament is essential because it provides a connection between alveolar bone and cementum and contains cells capable of forming and remodelling the three connective tissues of the periodontal complex [2]. There are various approaches available for periodontal regeneration, such as utilizing bone grafts, bone substitutes, guided tissue regeneration (GTR), growth factors and lastly tissue engineering techniques-either individually or in combination. Alloplasts offer a favorable alternative to allografts and xenografts due to their unlimited availability and lack of risk for disease transmission [3]. Among these, bioactive glass promotes bone formation through its ability to adsorb proteins essential for osteoblast function and mineralized matrix formation [4]. The putty form of bioactive glass, which incorporates glycerin and polyethylene glycol, enhances cohesiveness of the particles, thereby improving handling and reducing particle migration from the surgical site [5]. Histological studies also show that these particles can inhibit rapid epithelial growth [6, 7]. Growth factors are crucial in promoting periodontal tissue regeneration. The 2nd-generation platelet concentrate PRF, provides benefits over platelet-rich plasma (PRP). This fibrin matrix is adaptable and easy to suture, continuously releases growth-promoting proteins like platelet-derived growth factor (PDGF), vascular endothelial growth factor (VEGF), transforming growth factor-β (TGF-β), and matrix glycoproteins into the surgical site for up to seven days, as shown *in vitro* [8]. Combining a graft material with both osteoconductive and osteostimulatory properties with autologous PRF has the potential to support restoration and regeneration of periodontal structures in intrabony defects. Therefore, it of interest to compare the bioactive glass putty with and without platelet rich fibrin in the treatment of periodontal intrabony defects.

## Materials and Methods:

The study participants were taken from, Department of Periodontology, following the acquisition of their written informed consent. Clearance was obtained from the institutional ethics committee before initiating the study. The study included 32 intrabony defects from patients who were diagnosed with periodontitis. The split-mouth design required random assignment of each patient's defect site into control and test groups.

## Inclusion criteria:

[1] Subjects of both genders, between 20 and 60 years of age with periodontitis.

[2] Each study subject with at least two quadrants with intrabony defects.

[3] Study quadrant having at least one study site.

[4] Sites exhibiting PPD of ≥5 mm and a minimum CAL of 5 mm, assessed with UNC-15 probe

[5] Radiologically evident angular bone defects in the study site.

## Exclusion criteria:

[1] Presence of any documented systemic illness.

[2] Patients who are on anticoagulants.

[3] Patients with history of receiving any form of periodontal treatment in the last six months.

[4] Pregnant women or lactating women.

[5] History of smoking or any other form of tobacco use.

The defect assessment was performed through standardized intraoral periapical radiographs which were recorded using the paralleling technique [9]. A stationary reference point was chosen for recording the defects (*i.e.*, starting at CEJ and ending at apical extent of defect noted radiographically) at baseline and at 3, 6, and 9 months with the help of 1mm grid. The degree of defect fill was recorded. Changes in bone levels were analysed using standardized radiographic evaluations. The periodontal condition of each participant was evaluated through a comprehensive intraoral and periodontal examination. The following parameters were recorded which includes Plaque Index (PI), Gingival Index (GI) [10, 11], Probing Pocket Depth (PD) [12, 13] and Clinical Attachment Level (CAL) [14]. Initial treatment comprised of oral hygiene education, scaling and root planing utilising local anaesthesia with adjustment of the occlusion when required. The use of 0.2% chlorhexidine mouthrinse two times a day was recommended to patients for chemical plaque control. After four weeks, sites with persistent pocket depths ≥ 5 mm and radiological identification of angular bone loss were scheduled for flap surgery. Intrabony defects were arbitrarily allocated to any of the following: the control group (treated with bioactive glass putty only) or the test group (treated with a combination of bioactive glass putty with PRF). Antecubital vein was utilized for withdrawing 10 mL of venous blood into plain tubes without anti-clotting agents and spun at 3000 revolutions per minute for ten minutes and PRF was prepared [15]. The resulting clots were gently compressed using sterile gauze to create membranes. Under local aesthesia, reflection of a full-thickness mucoperiosteal flap was done, and debridement was performed at the test site. The bioactive glass putty (Novabone Putty) was packed into the defect from the base upward. PRF membrane was then placed and secured using 4 -0 Vicryl sutures. In the control site only, bioactive glass was compacted. After the procedure, the patient was prescribed antibiotics (amoxicillin 500 mg, 3 times per day for 5 days) along with analgesics (ibuprofen 400 mg, taken 2 times daily for 3 days). A non-eugenol dressing (Coe-Pack) was applied over the surgical site. The dressing was removed 7 days after the surgery.

## Results:

This study consisted of 16 subjects with 4 (25%) females and 12 males (75%). The subjects aged between 26-45 years. Data measured on a continuum was characterized as Mean ± SD. Statistical analysis was carried out using parametric tests, as the dataset conformed to a normal distribution. The independent t-test and paired t-test employed in assessing statistical significance between and within groups, respectively. Statistical analyses were executed in SPSS, with a p-value < 0.05 indicating significance. There was a significant reduction in the average probing depth score from 0 to 9 months in test group when compared to control group (p value <0.05) While evaluating PPD between the case and control groups, it was found that during 0-3 months, the average difference in the control group and test group was 3.1± 0.7 and 4.1± 0.1 respectively. From 0- 6 months, the control and test groups had an average difference of 3.4± 0.9 and 4.4± 0.8 respectively. Subsequently, during 0-9 months, the average difference in the control and test group was 3.7± 0.7 and 4.7± 0.1. The p-values during 0-3 months, 0-6 months, and 0-9 months when control and test groups were compared were, 0.003, 0.01, and 0.002 (Table 1 - see PDF) which was statistically significant. The correlation of clinical attachment level between the case and control group. It was found that during 0- 3 months, the average difference between the control group and test group was 3.1± 0.7 and 4.1± 0.9 respectively. During 0- 6 months, the control and test groups had an average difference of 3.4± 0.7 and 4.5± 0.7 respectively. Subsequently, during 0-9 months, the average difference in the control and test groups was 3.7± 0.7 and 4.7± 0.9. The p-values during 0-3 months, 0-6 months, and 0-9 months when control and test groups were compared were, 0.03, 0.01, and 0.01 (Table 2 - see PDF) which was statistically significant. In the correlation of bone regeneration between the case and control group it was found that during 0-3 months, the average difference between the control group and test group was 0.2 ± 0.4 and 0.4 ± 0.5 respectively. During 0- 6 months, the control and test groups had an average difference of 1.2 ± 0.5 and 2 ± 0.5 respectively. Subsequently, during 0-9 months, the average difference between the control and test group was 1.9 ± 1.02 and 3 ± 1.1. The p-values during 0-3 months, 0-6 months, and 0-9 months when control and test groups were compared were, 0.48, 0.006, and 0.007 respectively (Table 3 - see PDF). There was significantly higher bone regeneration at 6 and 9 months in the test group when compared to the control group. In the comparison of plaque index between the case and control group it was found that during 0-3 months, the average difference between the control subjects and test subjects was 2.3 ± 0.1 and 0.8 ± 0.07. During 0-6 months, the control and test groups had an average difference of 2.3 ± 0.1 and 0.6 ± 0.04 respectively. Subsequently, during 0-9 months, the average difference in the control and test groups was 2.3 ± 0.1 and 0.06 ± 0.2. The p-values during 0-3 months, 0-6 months, and 0-9 months when control and test groups were compared were, 0.001, 0.001, and 0.001 (Table 4 - see PDF) which was statistically significant. In the correlation of the gingival index between the case and control group it was found that during 0-3 months, the average difference between the control subjects and test subjects were 2.3 ± 0.07 and 0.7 ± 0.07 respectively. During 0- 6 months, the control and test groups had an average difference of 2.3 ± 0.07 and 0.6 ± 0.05 respectively. Subsequently, during 0-9 months, the average differences between the control and test groups were 2.3 ± 0.07 and 0.06 ± 0.04. The p-values during 0-3 months, 0-6 months, and 0-9 months when control and test groups were compared were, 0.001, 0.001, and 0.001 (Table 5 - see PDF) which was statistically significant.

## Discussion:

Periodontal therapy aims primarily to gain access to diseased sites, reduce pocket depths, halt the progression of periodontal disease, and restore lost periodontal structures. Over the years, various regenerative techniques have been employed to achieve the ideal outcome of true regeneration a key goal in periodontal therapy. However, no single method has emerged as a definitive standard due to limitations inherent to each approach. Bone graft is commonly utilized in periodontal regenerative procedures to support bone regeneration by filling osseous defects. Among the alloplastic materials studied, bioactive glass has demonstrated promising osteoconductive and osteostimulatory effects. In the current investigation, Novabone putty, easily moldable, pre-mixed composite containing bioactive calcium-phospho-silicate particles was utilized [16]. PRF, a 2nd-generation platelet concentrate, consists of very good levels of concentration of platelets and growth factors. Due to its unique physical and biochemical properties, PRF is especially effective in promoting wound healing in periodontal therapy. Based on these qualities, this study investigated the use of PRF as a regenerative aid in treating intrabony periodontal defects [17]. The goal of this research was assessment of the regenerative outcomes of Novabone putty used alone or along with a combination with PRF. A split-mouth approach was utilized to evaluate and compare clinical and radiographic results at treated areas in the same patient. Control sites received Novabone putty only, while test sites were treated with both Novabone putty and PRF. There was improvement in the two groups with statistical significance in plaque and gingival index scores, reflecting a reduction in inflammation from baseline (2.3 ± 0.1 and 2.3 ± 0.7) to 9 months post-treatment (0.06 ± 0.2 and 0.6 ± 0.04), as detailed in (Tables 4 and 5 - see PDF). Significant decrease in PPD and gain in clinical attachment levels and radiographic bone levels were observed in both groups, supporting previous findings by Zamet *et al.* (1997) [18]. The studies by Froum *et al.* (1998) [19] and Grover *et al.* (2013) [20] reported comparable regenerative results, strengthening the capability of bioactive glass in treating intrabony defects. Also, the combination of bioactive glass with other materials-such as e-PTFE membranes (Nevins *et al.* 2000; Yukna *et al.* 2001) [21, 22], bioresorbable membranes (Mengel *et al.* 2006) [23] and enamel matrix derivatives (Sculean *et al.* 2002; Kuru *et al.* 2006) have showed improved regenerative effects [24, 25]. PRF has also shown potential in treating furcation and intraosseous defects, as evidenced by studies from Bajaj *et al.* (2013), Sharma *et al.* (2011), Pradeep *et al.* (2012), and Ajwani *et al.* (2015) respectively [26, 27, 28-29]. Moreover, synergistic effects of PRF with various bone graft materials have been reported by Shah *et al.* (2015), Elgendy *et al.* (2015), Bansal *et al.* (2013), and Pradeep *et al.* (2012), indicating improved periodontal regeneration outcomes [30, 31-32]. However, contrasting results have been observed by Ong *et al.* (1998) [33] and Chacko *et al.* (2014) [7] who found only modest enhancements clinically and radiologically in their parameters when bioactive glass was used, attributing the limited success to factors such as particle size variation, inconsistent defect characteristics, and differing methodologies. Dybvik *et al.* (2007) also reported no significant benefits of bioactive glass over open flap debridement, likely due to the inclusion of severely compromised teeth with deep defects and mobility [34]. In the current study, defect sites treated with the PRF and Novabone putty combination demonstrated significantly better outcomes compared to sites treated with Novabone putty alone. Both groups started with mean baseline pocket depths (7.14 mm for Novabone and 7.43 mm for PRF + Novabone), and both showed reductions over time. By the 6-month, the control subjects exhibited a probing depth decrease of 3.67 mm, while the test subjects showed a decrease of 3.37 mm [29]. At 9 months, these reductions were 3.42 mm for the control subjects and 3.11 mm for the test subjects, respectively showing significant reductions in test group when compared to control group (Table 1 - see PDF). These results align with observations by Lekovic *et al.* in their PRF-BPBM group [35]. Clinical attachment level (CAL) gains were also more pronounced in the test subjects (4.7 ± 0.9 mm) compared to the control subjects (3.7 ± 0.7 mm) by the end of the study period (Table 2 - see PDF), again surpassing outcomes reported in comparative studies [35]. Radiographic assessments, carried out at baseline and at 3, 6, and 9 months, adhered to standardized projection techniques to reduce measurement errors, as recommended by Lang & Hill (1977) [36]. Prefabricated film holders helped maintain consistency in image capture. Bone changes were radiographically evident as early as three months post-treatment, in line with Gupta *et al.* observations that PRF, being an abundant reservoir of platelet-derived growth-promoting factors, may accelerate bone formation, supporting the rationale for early radiographic evaluations [37]. Both groups exhibited progressive radiographic bone fill over the study period. However, the test group consistently showed greater defect fill 0.4 ± 0.5 mm vs. 0.2 ± 0.4 mm at 3 months, 2 ± 0.5 mm vs. 1.2 ± 0.5 mm at 6 months, and 3 ± 1.1 mm against 1.9 ± 1.02 mm at 9 months (Table 3 - see PDF). The superior bone fill observed in the combination group aligns with findings by Lekovic *et al.* in similar regenerative contexts. Comparative analysis revealed superior bone regeneration in the PRF-Novabone cohort (3.0±1.1 mm defect reduction) versus Novabone alone (1.9±1.02 mm) at 9-month follow-up [35, 38]. In conclusion, we observed that combining PRF with Novabone putty resulted in improved clinical and radiographic outcomes for managing periodontal intrabony defects. These results suggest that PRF may serve as a valuable adjunct to traditional regenerative therapies and warrants further exploration in clinical practice.

## Conclusion:

32 intrabony periodontal defect sites across 16 patients were assessed using a split-mouth approach to compare the potency of bioactive glass putty (Novabone putty) alone versus its combination with platelet-rich fibrin (PRF). The test subjects administered with Novabone putty fused with PRF, showed an enhanced mean bone fill when correlated with the results of the control group that received Novabone putty alone. Multiple variables affect regenerative periodontal results including patient and defect selection, diagnostic accuracy, selected treatment interventions, and the follow-up period. Therefore, treatment goals must be established with a clear and practical awareness of these factors. Adding PRF to the bone graft material produced positive outcomes, indicating its ability to improve periodontal regeneration. It is recommended that future studies with bigger sample sizes need to be conducted to validate our results.
